# Focal aggregates of normal or near normal uveal melanocytes (FANNUMs) in the choroid: a distinct clinical and histopathological entity?

**DOI:** 10.1007/s00417-020-04851-0

**Published:** 2020-07-25

**Authors:** James J. Augsburger

**Affiliations:** Department of Ophthalmology, University of Cincinnati College of Medicine, Cincinnati, OH USA

**Keywords:** Choroidal nevus, Uveal melanocyte, Choroidal freckle, FANNUM (focal aggregate of normal or near normal uveal melanocytes)

## Abstract

**Purpose:**

To define, describe, and illustrate a previously unreported category of discrete melanotic choroidal melanocytic lesion.

**Methods:**

Prospective ophthalmoscopic study of the ocular fundi of 79 light-skinned persons 50 years of age or older not referred for any evident fundus lesion, with detection of all evident discrete melanotic choroidal lesions > 0.3 mm in largest basal diameter.

**Results:**

One or more discrete dark-brown to gray choroidal lesions > 0.3 mm in largest basal diameter were detected in 27 of the 79 evaluated subjects (34.2%). All but four of the detected lesions were “flat” by both ophthalmoscopy and ultrasonography. A single flat lesion was present in one eye of 14 subjects whose fellow eye was normal, 2 or more flat lesions were evident in one eye of 5 subjects whose other eye was normal, and one or more lesions were evident in both eyes of 6 subjects.

**Conclusion:**

While some of the discrete small, flat melanocytic choroidal lesions detected in this study might have been choroidal nevi, the author hypothesizes that an indeterminate proportion of them may have been focal aggregates of normal or near normal uveal melanocytes (FANNUMs).

## Introduction

Most ophthalmologists know (or think they know) what a choroidal nevus is and believe that they can recognize one when they see it. The classic histopathological definition of a choroidal nevus elaborated by the late Lorenz Zimmerman is “a choroidal tumor composed of *atypical* but *benign* uveal melanocytes”[1]. Because clinicians cannot ascertain whether the component uveal melanocytes of an observed melanocytic choroidal lesion are benign or malignant and, if benign, normal, or atypical, they use clinical criteria (including thickness and largest basal diameter of the lesion and ophthalmoscopically evident features of the lesion) to diagnose a given melanotic posterior uveal melanocytic lesion as a nevus or alternative type of lesion. Most clinical definitions of a choroidal nevus include a statement indicating the maximal allowable size of the lesion (usually about 5 mm in largest basal diameter [2] but up to 10 mm according to some authors [3] and usually about 1 mm in thickness [3] but up to 3 mm in some cases [4]). Most clinical definitions also specify that the lesion may be as thin as “flat” [5] or “too slight to measure” [6]. A few definitions specify a minimal largest basal diameter required for classification as a choroidal nevus (from as small as 0.35 mm [7] or 0.5 mm [8] to as large as 1.5 mm [5, 9]); interestingly, none of the authors who mention a minimal required lesion diameter for diagnosis indicate how they would classify a discrete melanotic choroidal lesion smaller than the specified dimension. A few clinical definitions mention features such as “replacement of the normal choroidal architecture” and “obscuration of some of the choroidal blood vessels” by the choroidal lesion [9]. Some authors use different diagnostic criteria for choroidal nevi in different reported studies.

Given the different diagnostic criteria for choroidal nevi used by various investigators, it should come as no surprise that independent studies attempting to determine the prevalence of choroidal nevi have yielded grossly disparate values (Table 1). While part of the difference in choroidal nevus frequency determined by various investigators is likely to be due to inconsistent diagnostic criteria, at least some of the reported differences are undoubtedly due to differences in study populations and different methods of lesion identification used by the various investigators. For example, Hale and coworkers counted all discrete posterior uveal melanocytic lesions dark enough to be detected by transillumination in an autopsy eye study [10]. That study found a posterior uveal nevus in 6.5% of evaluated eyes. In the Blue Mountains Eye Study, the method of lesion detection used was evaluation of white light fundus photographic slides of the posterior fundus obtained using a standard fundus camera [8]. That study found a choroidal nevus in 6.5% of evaluated subjects. In studies of light-skinned individuals by Gass, the method of detection used was comprehensive fundus examination using indirect ophthalmoscopy [5, 11]. Surprisingly, Gass claimed to have detected a choroidal nevus in 29.0% of all evaluated persons and in 33.9% of those over the age of 50 years (Table 2). In spite of the wide range of frequencies of choroidal nevi shown in Table 1, the most recently published edition of the American Academy of Ophthalmology’s Basic and Clinical Science Course indicates (without any qualifying comments) that “choroidal nevi may occur in up to 8% of the population” [12]. To our knowledge, no investigators other than Gass have ever reported frequencies of choroidal nevi over 20% in prospective studies. In spite of this, this author is unaware of any published challenges to Gass’s findings.

**Table 1:** Comparison of reported prevalence of choroidal nevi in several commonly cited studies

Investigators (year)	Study type	Number of subjects	Percent
Ganley and Comstock (1973) [6]	Prospective DO/IO study	9 of 287 persons	3.1
Sumich et al. (1998) [8]	Prospective fundus photographic survey	232 of 3583 persons	6.5
Hale et al. (1965) [10]	Autopsy study	13 of 200 eyes	6.5
Gass (1974) [5]	Clinical experience	Unspecified	20.0
Gass (1977) [11]	Prospective IO study	73 of 250 persons	29.2

*DO* direct ophthalmoscopic, *IO* indirect ophthalmoscopic

**Table 2:** Frequency of choroidal nevi reported by Gass in prospective ophthalmoscopic study of 250 persons [11]

Age subgroup	Frequency	Percent
≤ 30 years	0 of 23	0.0
> 30 to ≤ 40 years	2 of 14	14.3
> 40 to ≤ 50 years	8 of 27	29.6
> 50 years	63 of 186	33.9

The author arranged to perform a prospective indirect ophthalmoscopic study of light-skinned persons with relatively light-colored irides without any previously recognized fundus abnormalities; all of whom were 50 years of age or older to determine whether he could confirm or refute Gass’s reported findings of the frequency of small melanocytic choroidal lesions. The study described in the following paragraph was reviewed and approved by the Institutional Review Board of the University of Cincinnati College of Medicine.

## Patients and methods

The author recruited volunteer subjects (most of whom were spouses of patients referred to the Ocular Oncology Service) and patients referred to the Ocular Oncology Service because of an epibulbar lesion for comprehensive fundus examination of each eye by indirect ophthalmoscopy. All patients had to be ≥ 50 years of age, be light-skinned (grades I–III on the Fitzpatrick skin phototyping scale) [13], and have relatively light-colored irides (grade 1 or 2 on the Massachusetts Eye and Ear Infirmary iris color grading scale)[14]. All patients were informed about the purpose of the study and consented to participate in the study. All patients agreed to undergo pupillary dilation of both eyes using one drop each of topicamide 1% and phenylephrine 2.5%. Each patient was examined by the author, who attempted to identify every discrete melanotic choroidal lesion > 0.3 mm in diameter (approximately 1/5th disc diameter) in each eye. The position and largest basal diameter of each identified fundus lesion were documented on a standard fundus drawing chart of the right or left eye. Every patient in whom a discrete melanotic choroidal lesion was identified underwent further evaluation by contact B-scan ultrasonography (performed by JJA) to determine whether any measurable thickening of the choroid (relative to the thickness of the surrounding normal choroid) could be detected. The author tabulated and summarized the collected information on these patients.

## Results

The author recruited 79 subjects to this study. Forty-three subjects were spouses of patients referred to the Ocular Oncology Service and 36 were patients referred to the Ocular Oncology Service for evaluation of an epibulbar lesion. Forty-seven subjects were women and 32 were men. As required by the study inclusion criteria, all of the subjects were ≥ 50 years of age, were light-skinned, and had relatively light-colored irides. The mean age of the 79 evaluated subjects was 64.3 years (range 50+ to 83 years).

At least 1 discrete melanotic choroidal lesion > 0.3 mm in largest basal diameter was identified in 27 of the 79 subjects (34.2%) (Table 3). A total of 54 discrete melanotic choroidal lesions were identified in the 27 affected individuals. Four of the 79 subjects (5.1%) had one lesion in one eye that was elevated slightly and exhibited prominent surface drusen but no clumps of orange pigment or associated serous subretinal fluid. Each of these lesions was categorized as a classic choroidal nevus. These four choroidal nevi ranged in size from 4.0 to 6.5 mm in largest basal diameter (mean 4.9 mm) and from 0.5 to 1.2 mm in maximal thickness (mean 0.8 mm).

**Table 3:** Frequency of flat melanotic choroidal lesions > 0.3 mm in largest basal diameter in current study of 79 subjects

One or more lesions in at least one eye	25 of 79	31.6%
One lesion in one eye	14 of 25	56.0%
Two or more lesions in one eye	6 of 25	24.0%
One or more lesions in both eyes	5 of 25	20.0%

**Fig. 1:**
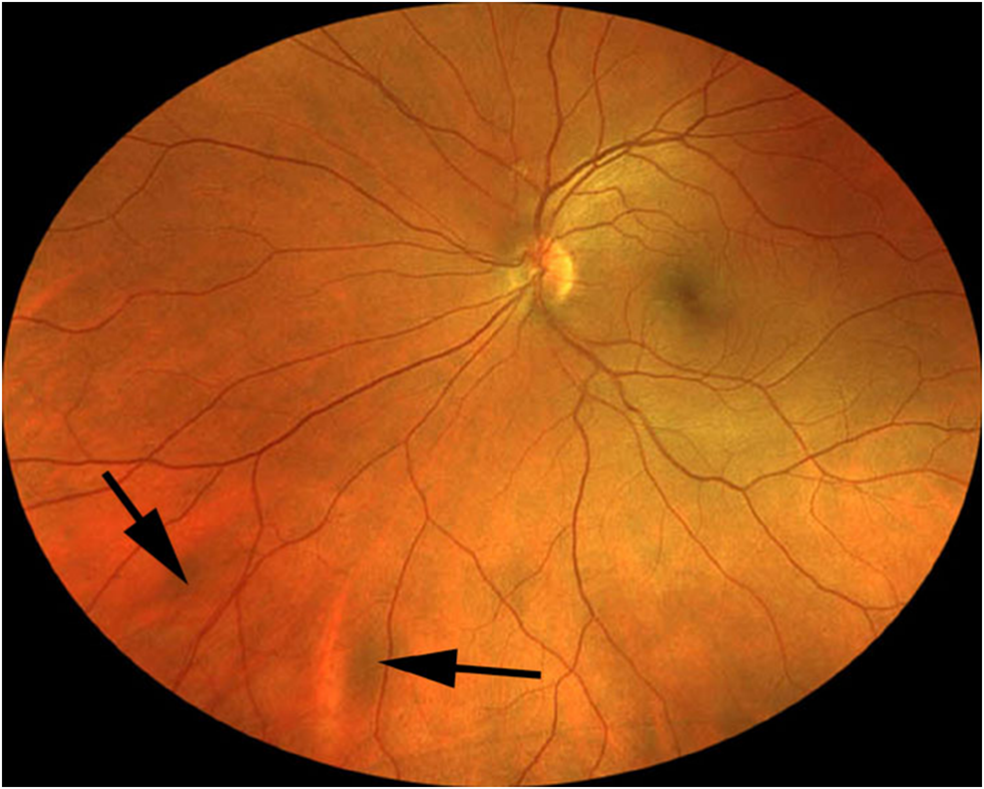
Two small melanotic choroidal lesions (arrows) believed by the author to be focal aggregates of normal or near normal uveal melanocytes. Both of these lesions are located adjacent to large choroidal veins inferonasally in the left eye

**Fig. 2:**
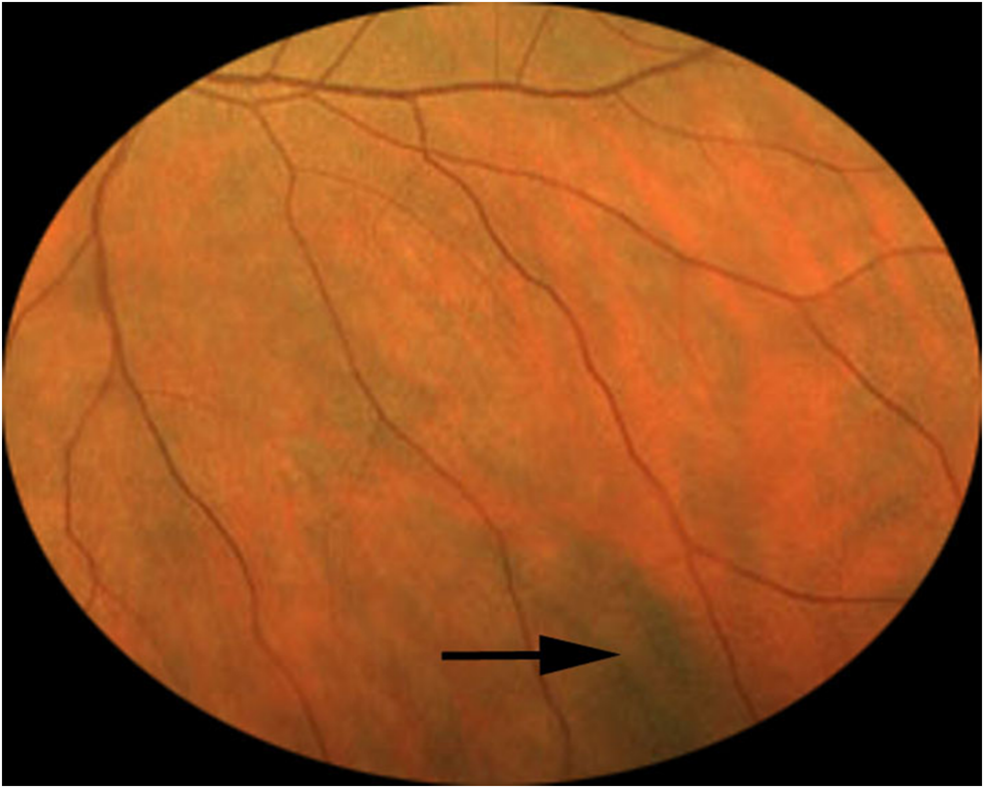
Solitary melanotic choroidal believed by the author to be a focal aggregate of normal or near normal uveal melanocytes. This lesion is located adjacent to a vortex vein ampulla inferotemporally in the left eye

**Fig. 3:**
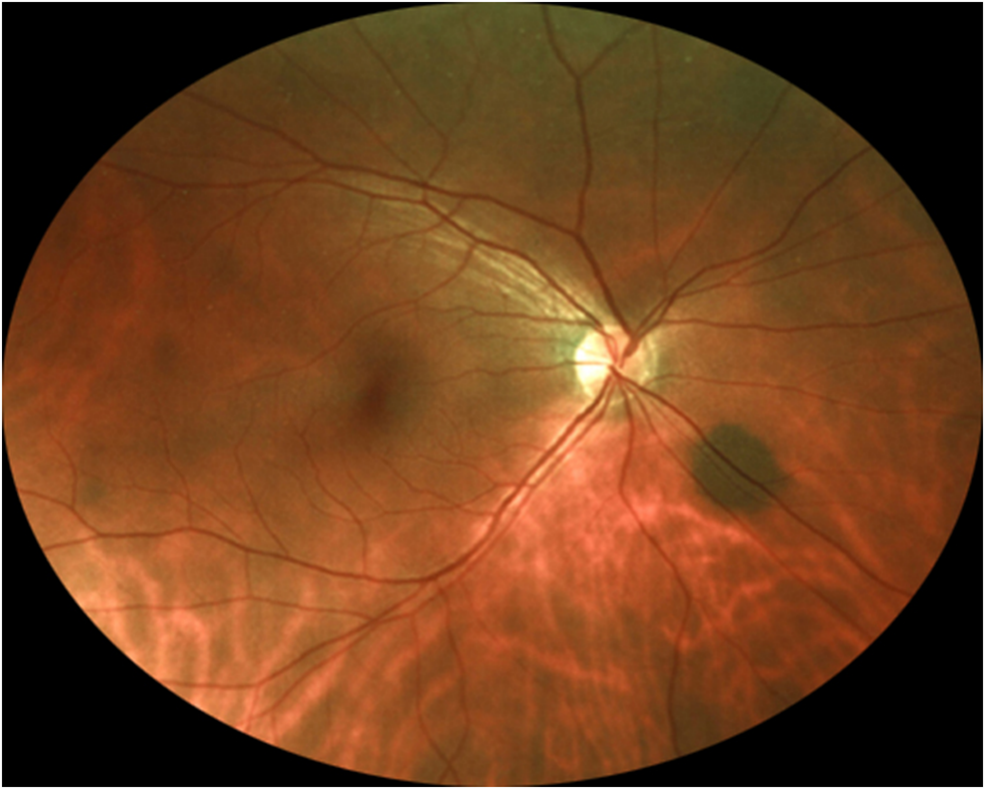
Solitary melanotic choroidal lesion believed by the author to be a focal aggregate of normal or near normal uveal melanocytes. This lesion is located just inferonasally from the optic disc in the right eye

**Fig. 4:**
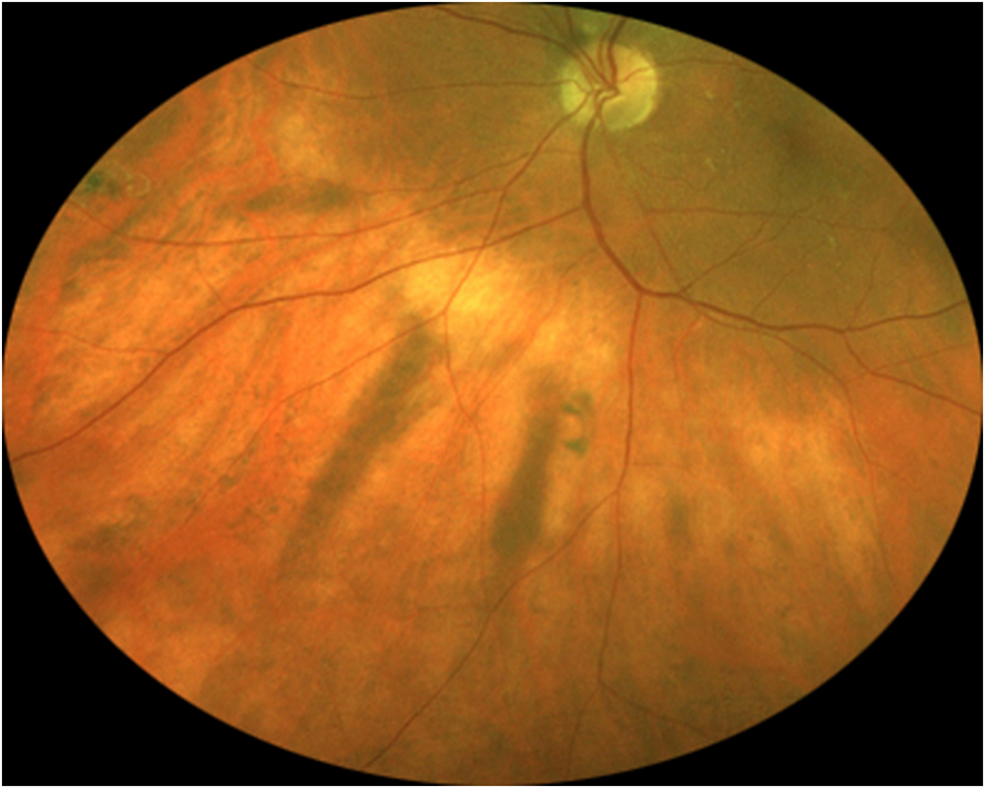
Two parallel ribbon-like melanotic choroidal lesions believed by the author to be focal aggregates of normal or near normal uveal melanocytes. These lesions are located in the fundus midzone inferonasally in the left eye. The position and orientation of these ribbon-like lesions suggest aggregation of uveal melanocytes along radial choroidal nerves

**Fig. 5:**
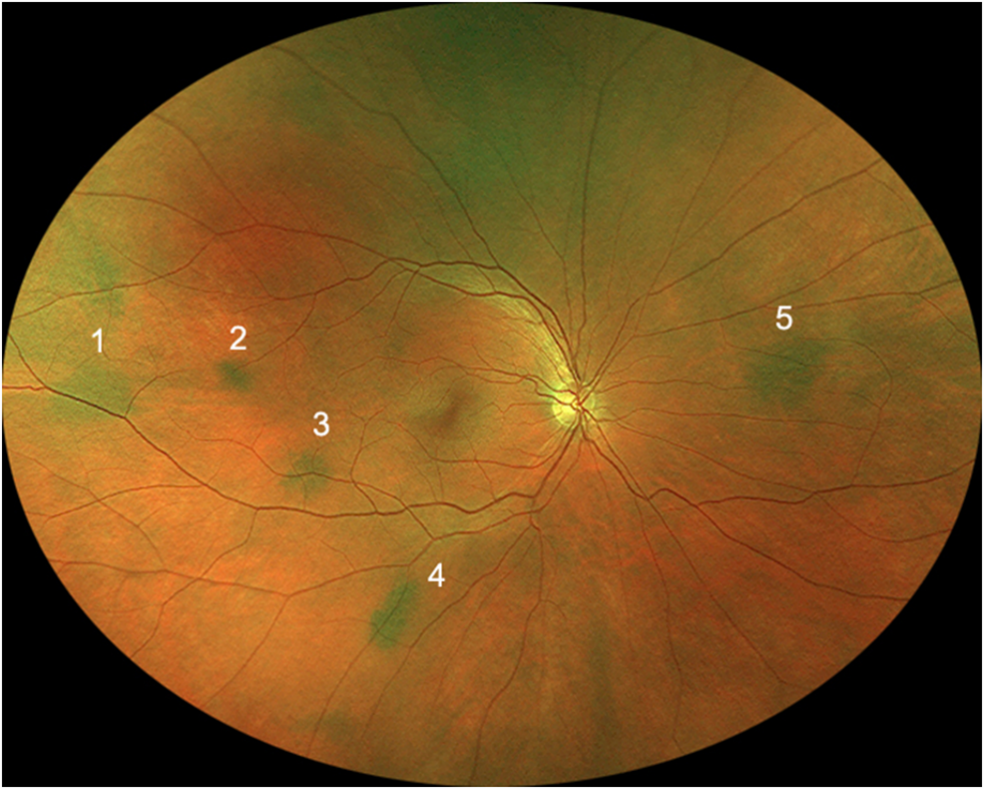
Right eye fundus of one study subject showing 5 discrete melanotic choroidal lesions believed by the author to be focal aggregates of normal or near normal uveal melanocytes.

Twenty-five of the 79 evaluated subjects (31.6%) had at least one discrete melanotic choroidal lesion that was not measurably thicker than normal choroid at that site by B-scan ocular ultrasonography (Figs. 1, 2, and 3). One of these 50 lesions was identified in a subject who also had a choroidal nevus in that eye, and another of these lesions was identified in the contralateral eye of a subject who had a choroidal nevus. These 50 lesions ranged in size from 0.35 to 6.0 mm in largest basal diameter (mean 2.1 mm) and from 0.35 to 3.0 mm in smallest basal diameter (mean 1.3 mm). Forty-three of these 50 lesions were at least 1 mm in largest basal diameter. One subject had two parallel radially oriented ribbon-like melanotic choroidal lesions (Fig. 4), the larger of which measured approximately 6 mm in length but was only about 1 mm wide. No other lesions in this category were more than 4 mm in largest basal diameter. With the exception of ribbon-like lesions, all other lesions were ovoid to geographic in basal shape. A single flat melanotic choroidal lesion was present in one eye only of 14 of the 25 affected persons (56%), two or more such lesions were identified in one eye only (Fig. 5) of 5 of the 25 affected persons (20%), and one or more lesions of this type was identified in both eyes of 6 of the 25 persons (24%) (Table 3). The most lesions of this type identified in any subject in this series was nine (5 in the right eye, 4 in the left). The most common locations for these flat melanotic choroidal lesions, noted for 39 of the 50 discrete flat melanotic choroidal lesions, were adjacent to prominent choroidal veins in the fundus midzone (Fig. 1) or adjacent to a vortex vein ampulla in the equatorial zone (Fig. 2). All 50 of these lesions were less prominent or invisible on green filter (red free) fundus photographs and fundus autofluorescence images than they were on color fundus photos, and all were accentuated on red filter fundus photographs.

## Discussion

The prevalence of discrete melanotic choroidal lesions in this population of light-skinned subjects with relatively light-colored irides (34.2%) is quite similar to the frequency of such lesions reported by Gass (33.9%) [11]. One might conclude from this study that its results confirmed Gass’s statement that choroidal nevi are present and evident in about 30–33% of light-skinned persons over the age of 50 years. However, only 4 of the 54 discrete melanotic choroidal lesions detected in the current series were measurably thicker than the surrounding normal choroid by ultrasonography. While these four slightly thickened choroidal lesions were almost certainly choroidal nevi, the question that should arise from this study (and Gass’ study) is whether most of the other melanotic choroidal lesions detected in this series (and many of the lesions counted in Gass’ series) were really nevi. If these lesions were not small choroidal nevi, then what were they? This author hypothesizes that many if not most of the discrete small, flat melanocytic choroidal lesions identified in this study (and in Gass’ prior study) may have been focal aggregates of normal or near normal uveal melanocytes (acronym FANNUM, plural FANNUMs) and not small choroidal nevi.

Normal uveal melanocytes comprise a broad spectrum of cells, including ones having dendritic, stellate, fusiform, and plump (polyhedral) morphological shapes that are distributed widely throughout the choroid [1]. Some normal uveal melanocytes contain a limited amount if any intracytoplasmic melanin, while others are densely packed with melanin granules. The one uniform characteristic of normal uveal melanocytes is the presence of a benign-appearing, centrally located nucleus with an absent or inconspicuous nucleolus in each cell. The uveal melanocytes that contain the most intracytoplasmic melanin tend to be more rounded and plump and have less prominent processes than do lightly pigmented or non-pigmented cells. The factors determining the amount of melanin that accumulates in an individual uveal melanocyte are largely unknown. What is known is that normal uveal melanocytes generally do not start to produce melanin until relatively late in embryologic life, and even at birth the cytoplasm of uveal melanocytes usually contains relatively little melanin [1]. As the individual ages, more melanin tends to be produced by the uveal melanocytes and accumulates within the cytoplasm of those cells. Unknown factors related to the local milieu of the choroid or minor non-neoplastic mutations that occur within a clone of choroidal melanocytes [15] may explain why some of these cells accumulate more intracytoplasmic melanin than others and why localized clusters of such hyperpigmented uveal melanocytes ultimately become evident ophthalmoscopically in some persons. In any event, age-related accumulation of intracytoplasmic melanin probably accounts for why most of the discrete small, flat melanocytic choroidal lesions of the type described in this report do not become evident ophthalmoscopically until adulthood.

In addition, normal uveal melanocytes are not fixed in position within the uveal stroma. Migration of normal uveal melanocytes into aggregates around or adjacent to larger choroidal blood vessels may account for at least some of the lesions encountered in this study. Because the normal choroid is substantially thicker than a single normal uveal melanocyte, FANNUMs in the choroid can be several cells thick without expanding the choroid’s thickness measurably. Hyperplasia (localized non-neoplastic proliferation) of choroidal melanocytes focally, stimulated by local environmental conditions or by minor non-neoplastic mutations affecting a limited clone of these cells [15], could also account for some discrete small, flat melanocytic choroidal lesions.

When focal aggregates of moderately to densely pigmented normal or near normal uveal melanocytes occur in eyes with relatively few pigmented uveal melanocytes in the surrounding choroidal stroma, these aggregates are likely to be visible ophthalmoscopically as flat melanotic choroidal lesions. In contrast, when multiple plump melanocytes filled with intracytoplasmic melanin are present throughout the choroid (as occurs in the eyes of dark-skinned individuals with dark brown irides and in the involved portion of the choroid in ocular melanocytosis [1]), the entire choroid appears dark gray to brown and focal aggregates of pigmented uveal melanocytes are unlikely to be detectable ophthalmoscopically. In individuals with intermediate cutaneous and iris pigmentation, some focal aggregates of normal or near normal uveal melanocytes containing cytoplasmic melanin are likely to be evident, albeit not as many as are likely to be evident in lightly pigmented eyes.

Although none of the lesions in the current study was evaluated by enhanced depth imaging optical coherence tomography, such testing of focal flat melanocytic choroidal lesions has been reported [16]. Dolz-Marco and coworkers evaluated two small melanotic choroidal lesions similar in appearance to some of the ones reported in the current study. One of the lesions appeared as a hyperreflective lesion involving all cross-sectional levels of the choroid; however, the other lesion appeared as a localized hyperreflective plate shadowing the sclera but not involving the middle or inner layers of the choroid. Neither of these lesions thickened the involved choroid appreciably compared with adjacent normal choroid. These observations suggest that the former lesion was either a FANNUM composed of densely melanotic uveal melanocytes distributed through the different layers of the choroid or a very small choroidal nevus or melanocytoma, while the latter lesion was almost certainly a FANNUM localized to the outer choroid-inner sclera at that site.

In 1966, Naumann and Zimmerman re-evaluated a series of choroidal melanocytic lesions that had been diagnosed histopathologically as benign choroidal nevi on initial pathological analysis at the Registry of Ophthalmic Pathology of the Armed Forces Institute of Pathology [17]. The author of the current article speculates that at least some of the choroidal lesions Naumann and Zimmerman included in their study group and reported may have been FANNUMs and not true nevi. If one accepts Zimmerman’s histopathological definition of a choroidal nevus as a tumor (i.e., a three-dimensional mass) composed of benign but atypical uveal melanocytes [1], then only those lesions thick enough to thicken the involved choroid measurably relative to adjacent normal choroid (i.e., large enough to be regarded as a tumor) should probably have been classified as true nevi, provided that the component cells were also “atypical but benign” uveal melanocytes. Focal lesions composed of plump uveal melanocytes containing a dense collection of melanin granules may thicken the choroid at that site slightly just as choroidal melanocytosis focally thickens the involved uvea. Because such cells represent part of the normal spectrum of uveal melanocytes [1], small melanocytic tumors composed of such cells that expand the choroid to a limited degree might also be regarded as a distinct subtype of FANNUM or small choroidal melanocytoma and not true choroidal nevi.

Some readers are likely to question why I am proposing the new term, FANNUM, for the focal small, flat melanocytic choroidal lesions encountered in this study and not simply calling them “choroidal freckles.” There are two reasons for this. First and foremost, the word freckle is already widely used in the dermatological literature, where it refers to a localized melanotic lesion of the skin due to an increased amount of melanin pigment within the affected melanocytes without any corresponding increase in the number of melanocytes. The second reason is the fact that the term choroidal freckle is already used (rather indiscriminately, this author believes) by many ophthalmologists to categorize virtually any small melanocytic choroidal lesion, including many unequivocally elevated choroidal nevi and even some small choroidal malignant melanomas. One needs only to survey the internet for photos classified as “choroidal freckle” to verify this point. Even the American Academy of Ophthalmology’s official website (https://www.aao.org) clouds this issue by posting a document entitled “Nevus (Eye Freckle).”

## Conclusion

It appears to me that most ophthalmologists and ophthalmic pathologists currently classify any discrete melanotic posterior uveal melanocytic lesion that is not a malignant melanoma as a choroidal nevus. In my opinion, such classification of all small melanocytic choroidal lesions represents a logical fallacy similar to George Callender’s classification of all melanocytic posterior uveal tumors that prompted enucleation as malignant melanomas [18]. I have hypothesized that many if not most discrete small, flat melanotic choroidal melanocytic lesions that are not malignant melanomas may be focal aggregates of normal or near normal uveal melanocytes (FANNUMs) and not true benign neoplasms (i.e., choroidal nevi). My hypothesis has pertinent relevance with regard to estimates of the frequency of “malignant transformation” of choroidal nevi into choroidal malignant melanomas [19].

This author urges ophthalmic pathologists who have access to histopathological tissue from autopsy eyes to identify discrete small, flat melanocytic choroidal lesions that may be present in them and study those lesions histopathologically (and possibly by cytogenetic and yet-to-be-developed technologies) in an attempt to determine whether the component uveal melanocytes are normal, near normal, or clearly abnormal and whether the lesions should be categorized as FANNUMs or choroidal nevi. I also urge them to reevaluate flat choroidal melanocytic lesions that they identified previously in enucleated eyes and categorized as choroidal nevi. Finally, I urge ophthalmologists who encounter discrete small, flat melanocytic choroidal lesions in their practices to document such lesions photographically (and possibly also by technologies such as optical coherence tomography). If an eye containing such a lesion comes to enucleation, hopefully the clinician will notify the ophthalmic pathologist about the lesion and encourage the pathologist to evaluate that lesion histopathologically.
